# Supplementary material for: Extracellular Vesicle-Serpine-1 Affects Neural Progenitor Cell Mitochondrial Networks and Synaptic Density: Modulation by Amyloid Beta and HIV-1
Source: Mol Neurobiol. 2023 Jul 17;60(11):6441–65. doi: 10.1007/s12035-023-03456-y (PMC10533645; doi:10.1007/s12035-023-03456-y)
Supplement: Supplementary file 1 — Supplementary file1 (PDF 1742 KB) [file 12035_2023_3456_MOESM1_ESM.pdf]

Extracellular vesicle-Serpine-1 affects neural progenitor cell mitochondrial networks and synaptic density: modulation by amyloid beta and HIV-1

Ibolya E. András, Nelson Serrano, Irina Djuraskovic, Nikolai Fattakhov, Enze Sun and Michal Toborek

Supplementary Material

Supplementary Figure 1. Serpine-1 and tPA activity in the released EVs (A,B). Non-transfected HBMEC were exposed to HIV-1 (30 ng p24/ml) and/or 100 nM Aβ (1-40) for 48 h. EVs were isolated from the culture media. A) Serpine-1 and B) tPA activities were determined by specific activity assays in the presence or absence of PAI039. Activity values were normalized to cell culture media volume (left graphs) or to EV protein content (right graphs). Values are mean ± SEM, n=4-5. One-, two- and three-way ANOVA with Šídák's and Tukey's multiple comparisons tests. *Statistically significant at $p<0.05$, **$p<0.01$. **(C) Oxygen consumption rate (OCR) and extracellular acidification rate (ECAR) in NPCs after PAI039 exposure**. NPCs were treated for 24 h with PAI039 (2 μM). OCR (upper graph) and ECAR (lower graph). Values are mean ± SEM, n=8. **Mitochondrial functions in recipient NPCs after exposure to HBMEC-derived EVs (D-L).** Non-transfected HBMEC were treated as in Figure 3. EVs were isolated from the culture media and employed for NPC treatment for 24 h in the presence or absence of the Serpine-1 inhibitor PAI039 (2 μM). (D) Seahorse Mitochondrial Stress Test diagram. (E) Oxygen consumption rates (OCR) and (F) Extracellular acidification rates (ECAR) in the treatment groups. (G) Non-mitochondrial oxygen consumption, (H) Basal respiration, (I) Maximal respiration, (J) Proton leak, (K) ATP production, (L) Spare respiratory capacity in the treatment groups are represented as % of the control. Representative graphs from three experiments. Values are mean ± SEM, n=6-18. One-, two- and three-way ANOVA with Šídák's and Tukey's multiple comparisons tests. *Statistically significant at $p<0.05$, **$p<0.01$, ***$p<0.001$.

Supplementary Figure 2. Impact of HBMEC-derived EVs on synaptic protein expression in NPCs (related to Figure 7). Non-transfected HBMEC were treated with HIV and/or Aβ and EVs were isolated as in Supplementary Figure 1; Then, human NPCs were exposed to HBMEC-derived EVs for 24 h, with selected cultures additionally treated with 2 μM PAI039 (P) as in Supplementary Figure 1. Synaptophysin (green; arrow heads) and PSD95 (red) immunoreactivity (arrows) as imaged by confocal microscopy. DAPI staining (blue) visualizes the NPC nuclei. The combined z-stack images with maximum intensity projection are representative from three experiments. Scale bar: 10 μm.

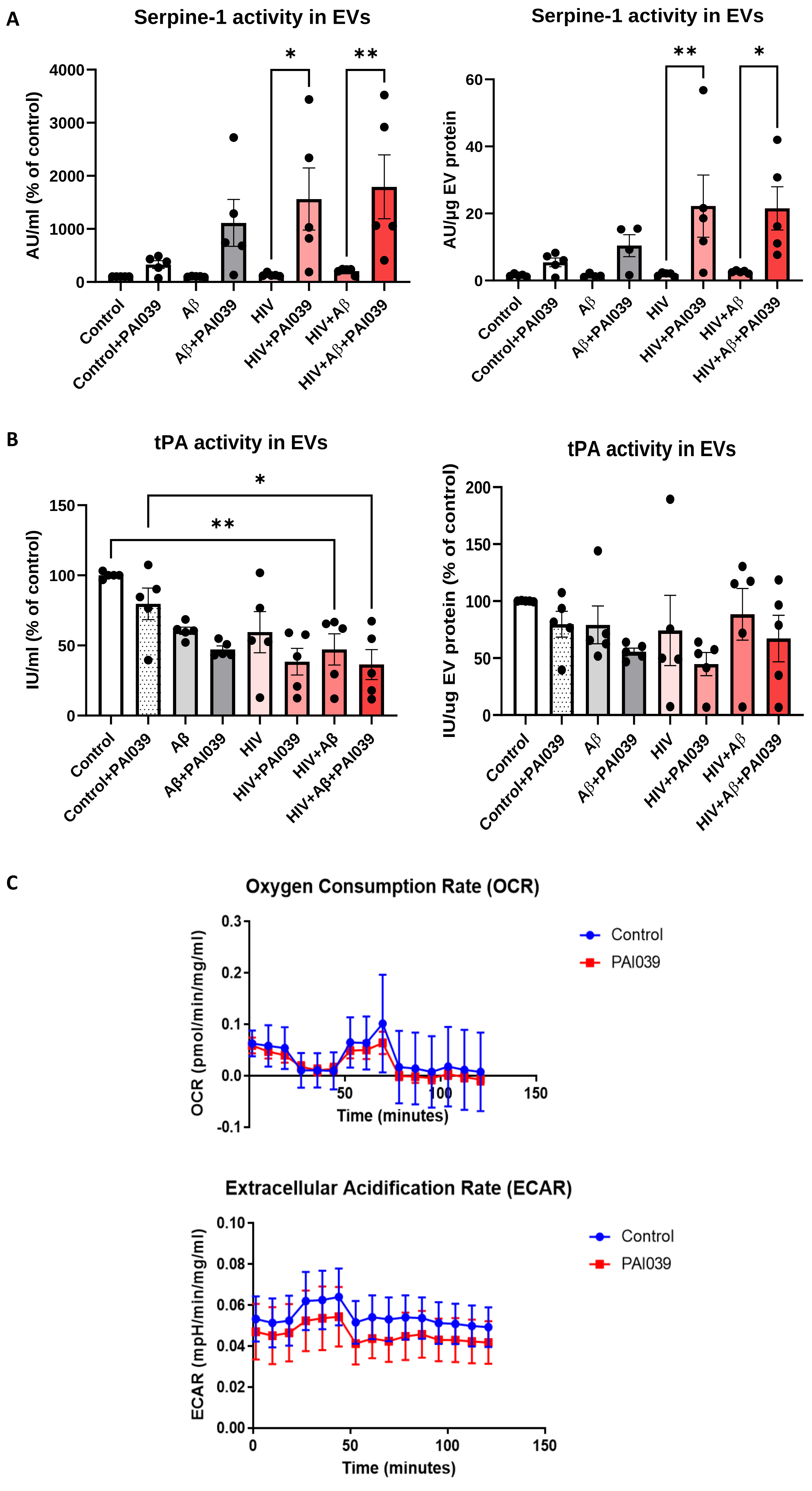
A
Serpine-1 activity in EVs
AU/ml (% of control)
4000
3000
2000
1000
0
*
**
Control
Control+PAI039
Aβ
Aβ+PAI039
HIV
HIV+PAI039
HIV+Aβ
HIV+Aβ+PAI039
Serpine-1 activity in EVs
AU/μg EV protein
60
40
20
0
**
*
Control
Control+PAI039
Aβ
Aβ+PAI039
HIV
HIV+PAI039
HIV+Aβ
HIV+Aβ+PAI039
B
tPA activity in EVs
IU/ml (% of control)
150
100
50
0
*
**
Control
Control+PAI039
Aβ
Aβ+PAI039
HIV
HIV+PAI039
HIV+Aβ
HIV+Aβ+PAI039
tPA activity in EVs
IU/ug EV protein (% of control)
200
150
100
50
0
Control
Control+PAI039
Aβ
Aβ+PAI039
HIV
HIV+PAI039
HIV+Aβ
HIV+Aβ+PAI039
C
Oxygen Consumption Rate (OCR)
OCR (pmol/min/mg/ml)
0.3
0.2
0.1
0.0
-0.1
50
100
150
Time (minutes)
Control
PAI039
Extracellular Acidification Rate (ECAR)
ECAR (mpH/min/mg/ml)
0.10
0.08
0.06
0.04
0.02
0.00
0
50
100
150
Time (minutes)
Control
PAI039

D

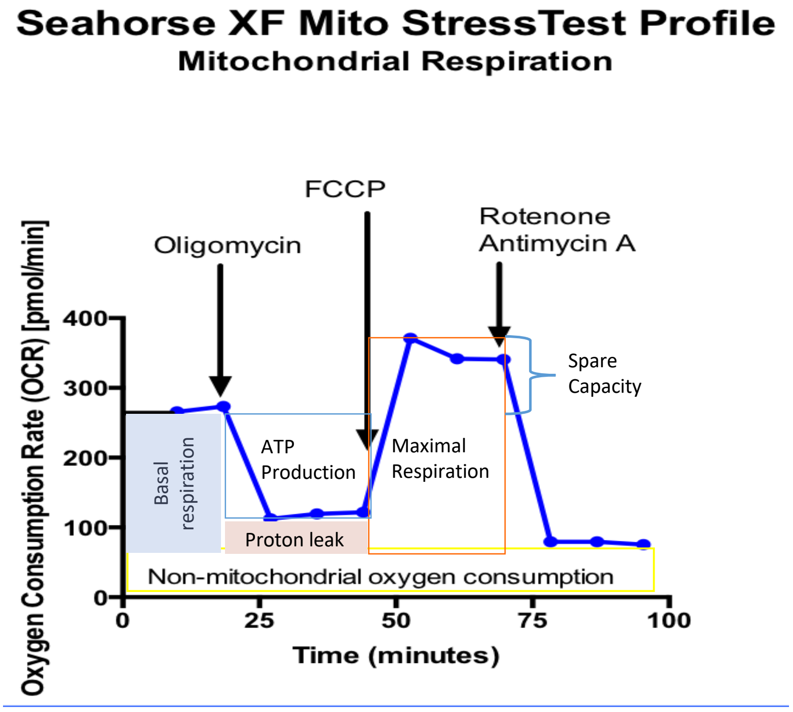

Seahorse XF Mito StressTest Profile
Mitochondrial Respiration
FCCP
Oligomycin
Rotenone
Antimycin A
Oxygen Consumption Rate (OCR) [pmol/min]
400
300
200
100
0
Spare
Capacity
Basal
respiration
ATP
Production
Maximal
Respiration
Proton leak
Non-mitochondrial oxygen consumption
0
25
50
75
100
Time (minutes)

E

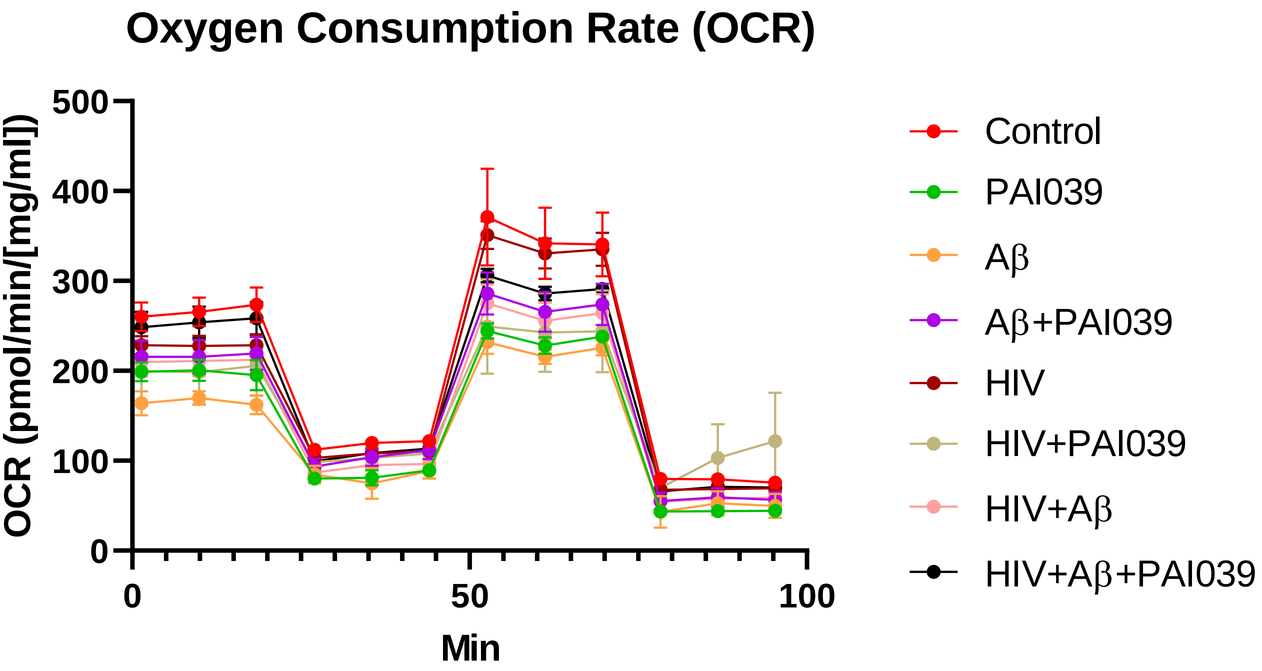

Oxygen Consumption Rate (OCR)
OCR (pmol/min/[mg/ml])
500
400
300
200
100
0
0
50
100
Min
Control
PAI039
Aβ
Aβ+PAI039
HIV
HIV+PAI039
HIV+Aβ
HIV+Aβ+PAI039

F

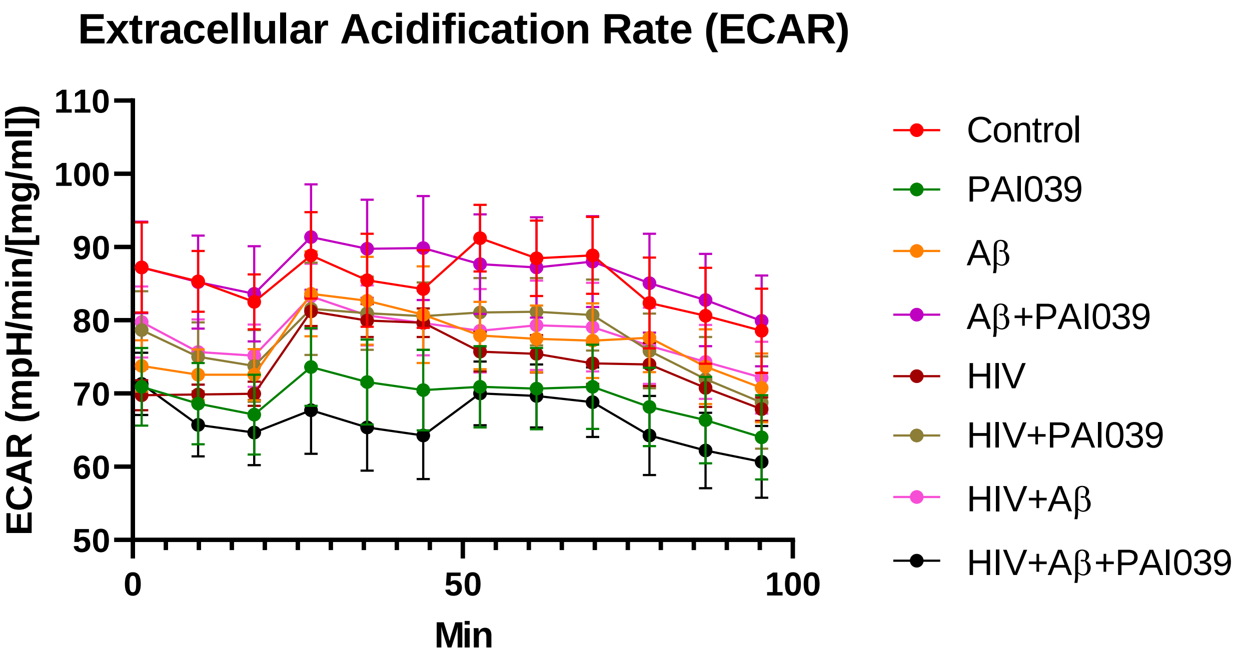

Extracellular Acidification Rate (ECAR)
ECAR (mpH/min/[mg/ml])
110
100
90
80
70
60
50
0
50
100
Min
Control
PAI039
Aβ
Aβ+PAI039
HIV
HIV+PAI039
HIV+Aβ
HIV+Aβ+PAI039

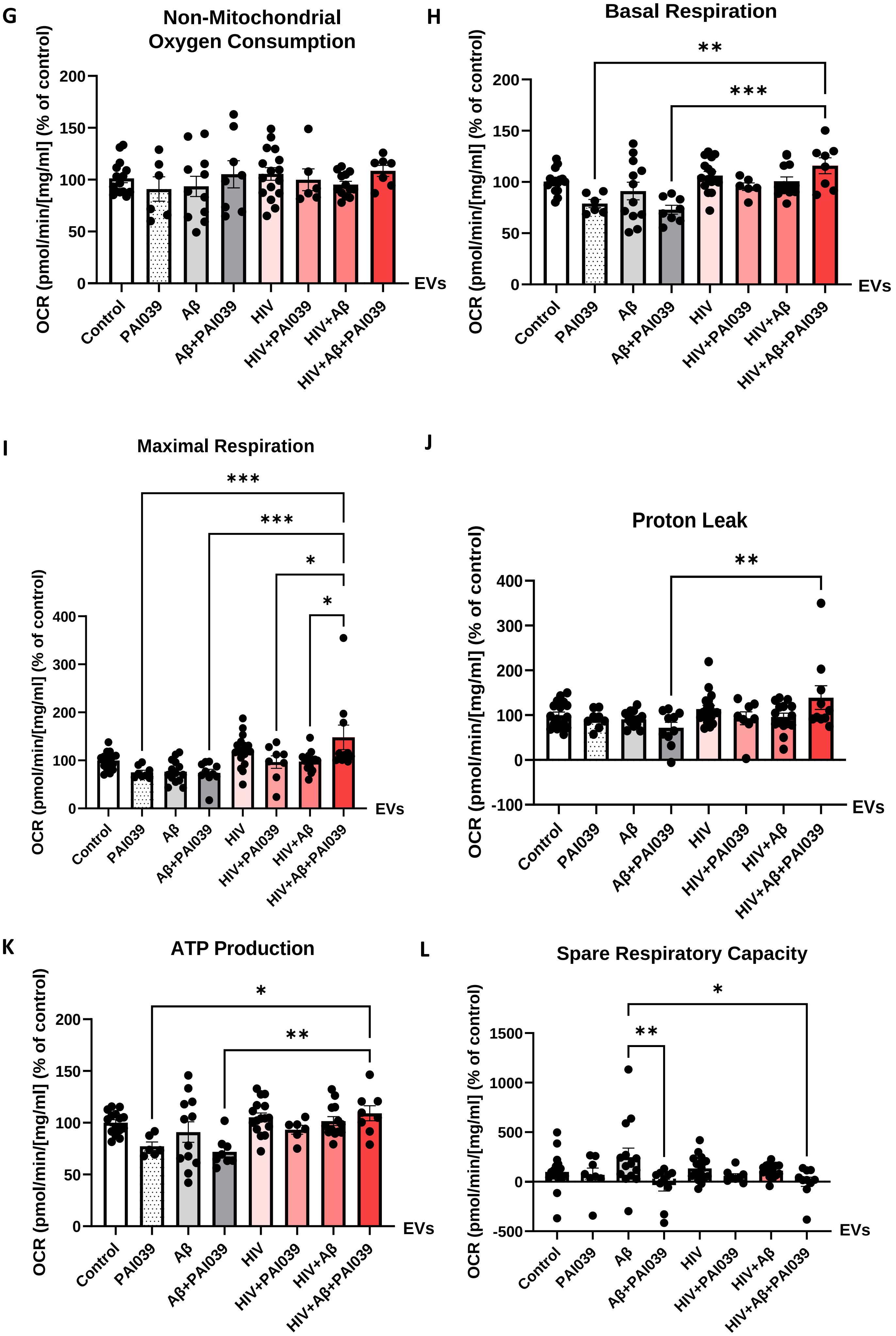

Supplementary Figure 1

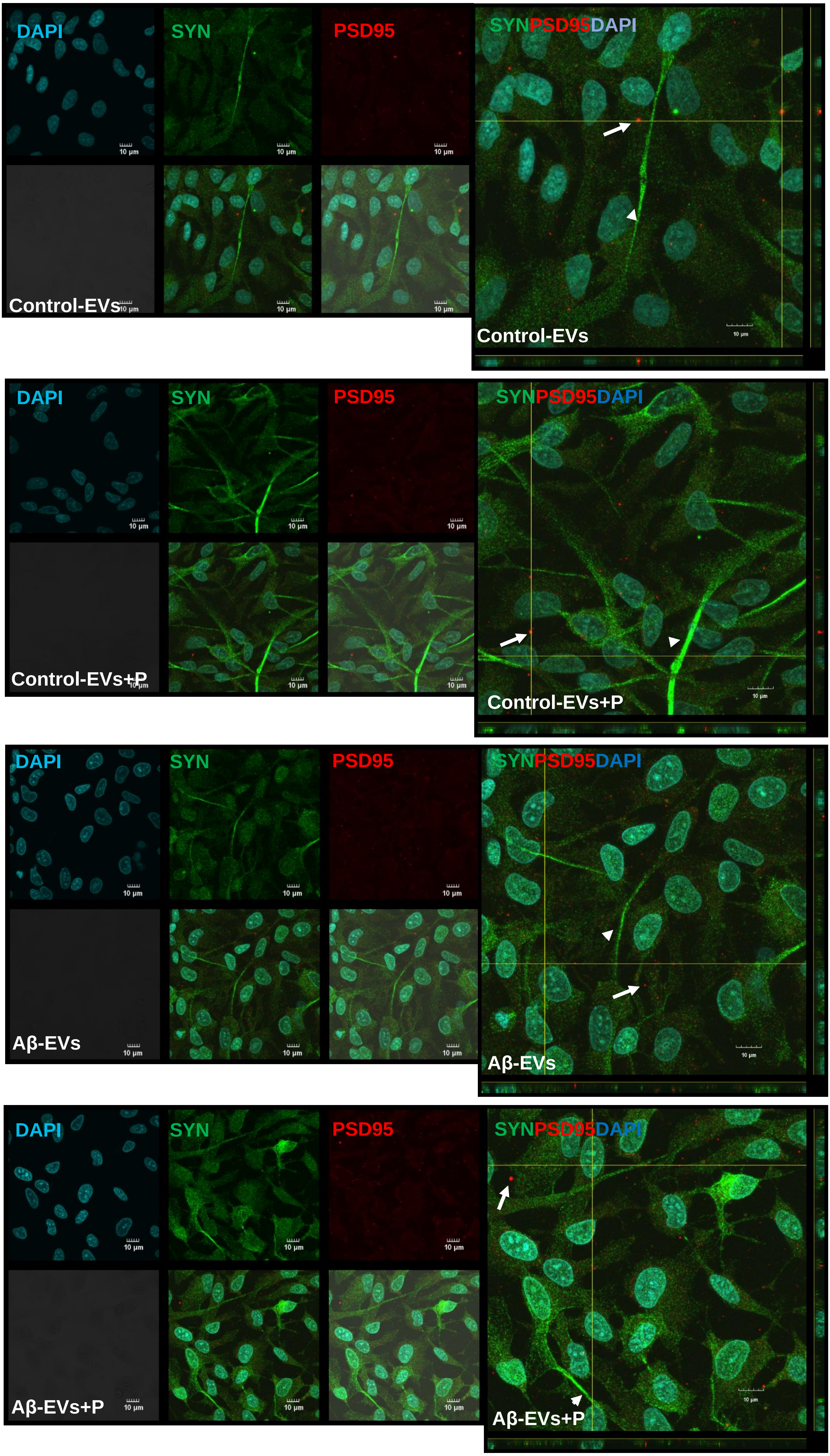
DAPI
SYN
PSD95
SYNPSD95DAPI
Control-EVs
Control-EVs+P
Aβ-EVs
Aβ-EVs+P
10 μm

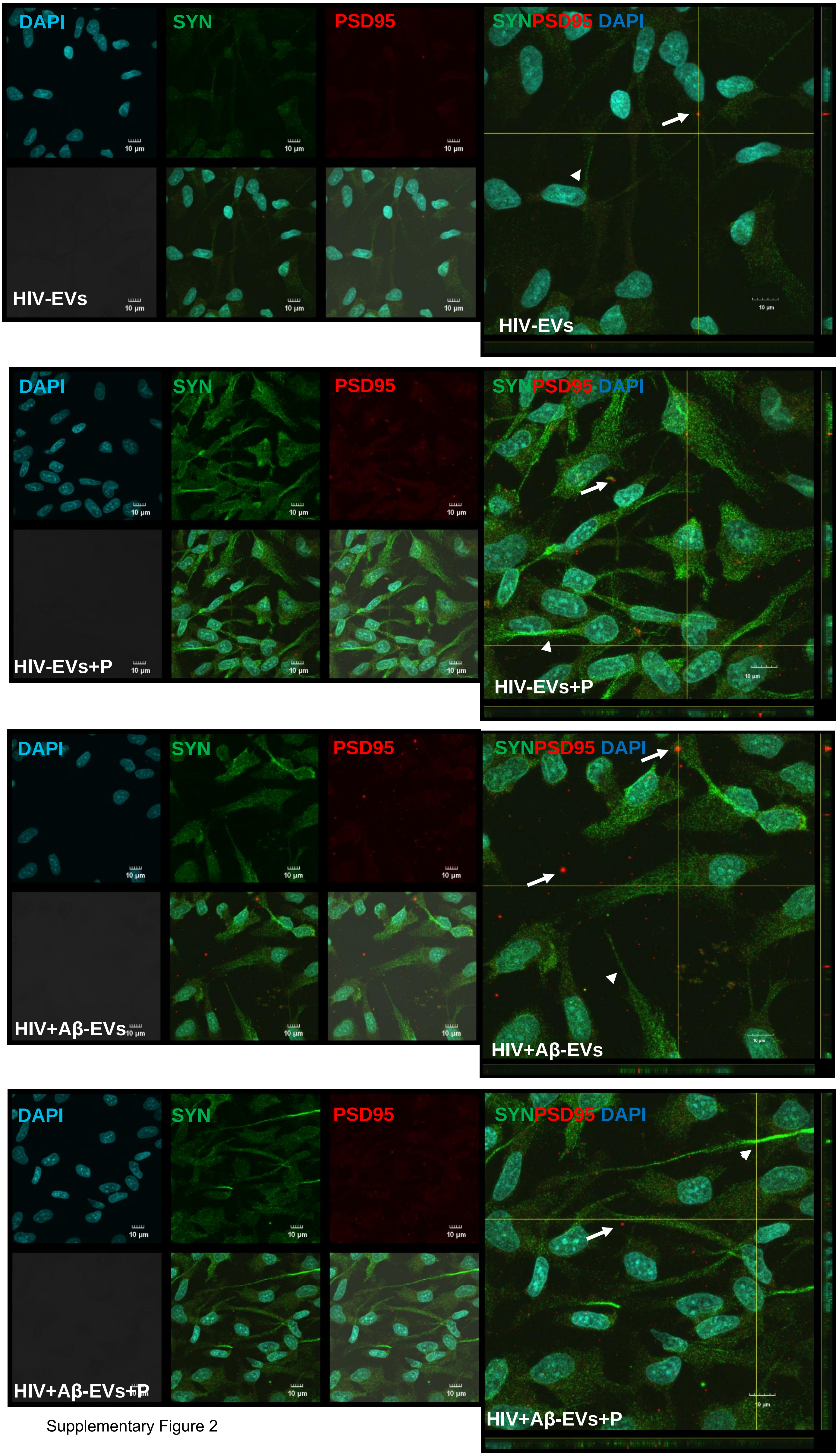

Supplementary Figure 2
